# Toward an animal model of Progressive Supranuclear Palsy

**DOI:** 10.3389/fnins.2024.1433465

**Published:** 2024-10-03

**Authors:** Priyanka, Syeda Hania Qamar, Naomi P. Visanji

**Affiliations:** ^1^Tanz Centre for Research in Neurodegenerative Diseases, University of Toronto, Toronto, ON, Canada; ^2^Department of Laboratory Medicine and Pathobiology, University of Toronto, Toronto, ON, Canada; ^3^Krembil Brain Institute, University Health Network, Toronto, ON, Canada; ^4^Edmond J. Safra Program in Parkinson's Disease and the Morton and Gloria Shulman Movement Disorders Clinic, Rossy Progressive Supranuclear Palsy Centre, Toronto Western Hospital, Toronto, ON, Canada

**Keywords:** Progressive Supranuclear Palsy, animal model, tau, sarkosyl-insoluble, inoculations, cytopathologies

## Abstract

Progressive Supranuclear Palsy (PSP) is a rare and fatal neurodegenerative tauopathy which, with a rapid clinical progression coupled to a strong degree of clinico-pathologic correlation, has been suggested to be a “frontrunner” in translational development for neurodegenerative proteinopathies. Elegant studies in animals have contributed greatly to our understanding of disease pathogenesis in PSP. However, presently no animal model replicates the key anatomical and cytopathologic hallmarks, the spatiotemporal spread of pathology, progressive neurodegeneration, or locomotor and cognitive symptoms that characterize PSP. Current models therefore likely fail to recapitulate the key mechanisms that underly the pathological progression of PSP, impeding their translational value. Here we review what we have learned about PSP from work in animals to date, examine the gaps in modeling the disease and discuss strategies for the development of refined animal models that will improve our understanding of disease pathogenesis and provide a critical platform for the testing of novel therapeutics for this devastating disease.

## Introduction

Progressive Supranuclear Palsy (PSP), the second most common tauopathy after Alzheimer's disease (AD), is a uniformly fatal neurodegenerative movement disorder with no existing treatment. Prevalence ranges from 1 to 18 per 100,000 persons, and median survival is ~5 years (Nath et al., 2001; Barer et al., 2022; Takigawa et al., 2016). PSP exhibits remarkable clinical heterogeneity, with nine distinct clinical subtypes currently recognized, the most common of which is PSP Richardson Syndrome (PSP-RS) accounting for up to 50% of cases (Höglinger et al., 2017). Core clinical features occur across four functional domains; ocular motor dysfunction; postural instability; akinesia and cognitive dysfunction (Höglinger et al., 2017). PSP represents a bleak diagnosis with no existing disease modifying therapies, though, as a primary tauopathy, with a rapid clinical progression coupled to a strong degree of clinico-pathologic correlation, it has been proposed to be a frontrunner in translational value amongst the tauopathies (Shoeibi et al., 2019). However, a major impediment to the development of novel treatments for the disease is the lack of an animal model with proven translational value.

Pathologically, PSP is characterized by an abnormal accumulation of microtubule associated protein tau, encoded by the *MAPT* gene (Goedert, 2003). In the human brain, alternative splicing of *MAPT* gives rise to the expression of six isoforms of tau, half of which have 3-microtubule binding repeat domains (3R) and half with 4-microtubule binding repeat domains (4R) (Goedert et al., 2024; Goedert, 2003). Tau aggregates in PSP are composed exclusively of the 4R tau isoform and the recent use of cryo-electron microscopy has demonstrated that aberrant PSP tau harbors a three-layered fold with a unique filament structure that distinguishes it from other 4R tauopathies (Shi et al., 2021). Furthermore, in contrast to the paired helical filaments that characterize AD-related tau, PSP tau forms straight, unbranched filaments (Arima, 2006). Although we don't have a full understanding of how these exclusive structural characteristics of PSP tau might influence the distinct cell type specific pathology found in PSP, it's plausible that the unique disease relevant tau conformations described in PSP might have some bearing on the interaction of PSP tau with certain populations of cells in distinct brain areas (Majumder and Dutta, 2024). Thus, the conformational and filamentous features of tau aggregates in PSP could contribute to the unique cell-specific distribution of pathology distinctive of PSP. Importantly, Disease-associated tau filaments have been demonstrated to possess the ability to act as an aggregation promoting seed which can recruit native tau molecules to induce their aggregation (Goedert et al., 2017; Hyman, 2023). Indeed, a key tenet of our present understanding of the pathogenesis of PSP is the ability of tau seeds to self-propagate, spreading cell-to-cell throughout anatomically connected brain regions as the disease progresses (Goedert et al., 2017).

In PSP, accumulation of 4R tau is found in both neuronal and glial cells in distinct brain regions (Kovacs et al., 2020). A recent multi-center study revealed that in all clinical subtypes of PSP: (1) tau accumulation commences in the pallido-nigro-luysian axis, prior to cortical areas, highlighting the importance of these regions in early pathogenesis and (2) vulnerable nuclei exhibit distinct cytopathologies. Early in the disease, the substantia nigra (SN) exhibits predominantly neurofibrillary tangles and threads, the globus pallidus (GP) exhibits neuronal pathology as well as oligodendroglial coiled bodies, and the caudate putamen (CPu) harbors astrocytic pathology (Kovacs et al., 2020). Furthermore, the tufted astrocyte is a defining feature exclusive to PSP (Yoshida, 2014). The spatiotemporal spread of these three cytopathologies throughout the brain has been described in six sequential stages which are subsequently translated into six neuropathological stages of diagnosis (Kovacs et al., 2020; Stamelou et al., 2021).

At a biochemical level, the brains of patients with tauopathies harbor a spectrum of tau species ranging from low molecular weight fragments of degraded tau, through monomeric forms to soluble and insoluble high molecular weight species (Hyman, 2023; Tarutani and Hasegawa, 2022; Tarutani et al., 2022). A sarkosyl resistant 33-kDa fragment distinguishes the disease from other 4R tauopathies (Taniguchi-Watanabe et al., 2016). In AD, studies have suggested that the molecular diversity of tau is a driver of clinical heterogeneity (Tarutani and Hasegawa, 2022; Dujardin et al., 2020). However, which conformers among the spectrum of soluble and insoluble tau are primarily responsible for the spreading of pathology throughout the brain is a topic of heated debate (Mate De Gerando et al., 2023; Stern and Selkoe, 2023; Mate de Gerando et al., 2023). Our recent work has also demonstrated considerable biochemical variability exists in PSP tau, both between the different brain regions of a given patient and among patients (Martinez-Valbuena et al., 2023). Furthermore, variability in the seeding activity of tau between patients was shown to correlate with differences in clinical course and with immune system involvement, suggesting that there may be molecular subtypes of PSP that can be distinguished at a biochemical level. Such molecular diversity clearly adds considerable complexity to the modeling for the disease.

In sum, PSP can be distinguished from other tauopathies at a clinical, neuroanatomical, cytopathological and biochemical level. To confer maximum translational value, an animal model of PSP should attempt to recapitulate these defining aspects of the disease, a grand challenge! Thus, an ideal animal model of PSP would: (a) exist on a background that mirrors the expression of all isoforms of tau expressed in the human brain, (b) mimic the three key early cytopathologies of all subtypes of PSP in the pallido-nigro-luysian axis, (c) recapitulate key structural and biochemical features of PSP-tau, (d) exhibit neurodegeneration and an accompanying locomotor behavioral phenotype (e) use methodologies that are both reproducible and scalable. Although complicated by the presence of three distinct cytopathologies, and significant neuropathologic diversity, emerging evidence suggests that, at least in PSP-RS, tau may follow a sequential deposition throughout the human brain (Kovacs et al., 2020). If future evidence emerges to support this observation, then models might additionally attempt to replicate the sequential spread of pathology to neuroanatomically relevant regions of the brain. As we will discuss in this review, many great strides have been made in attaining such a model, however there are also gaps in the current state-of-the-art which we hope future studies, capitalizing on both an increased understanding of disease pathogenesis and novel technologies, will address to aid the development of desperately needed treatments for this devastating disease.

## Learnings from existing animal models of PSP

Efforts related to modeling PSP in animals can be divided into five broad categories: lesion-based models; Transgenic animals; Viral Vector based models; Rodent inoculation studies and Studies in the non-human primate.

### Lesion-based models

Early cholinergic neuronal loss in the pedunculopontine tegmentum (PPTg) is a hallmark of PSP pathology (Hirsch et al., 1987; Jellinger, 1988). The PPTg has synaptic connections to several regions susceptible to PSP tau pathology, notably the pons, GP, CPu, subthalamic nucleus and SN. In rats, selective ablation of cholinergic neurons of the PPTg using diphteria-urotensinII neurotoxin induces loss of acoustic startle reflex (ASR) which offers a valuable paradigm to explore this poorly understood clinical phenomenon in PSP (MacLaren et al., 2014a). Furthermore, selective lesion of the PPTg results in dopaminergic neuronal loss in the SN, a region that mainly receives cholinergic innervations from the PPTg, accompanied by a progressive decrease in motor abilities over a period of 1 year (MacLaren et al., 2018). The authors attribute the dopaminergic neuronal loss to reduced cholinergic innervation of the SN and suggest that cholinergic input from the PPTg provides nigral neurons with critical trophic support. This work certainly provides a valuable tool to explore ASR loss in PSP, and the role of cholinergic neurons in nigrostriatal dopamine neuron function. However, the translational value of this model is somewhat limited by the highly selective nature of the lesion and lack of involvement of tau pathology.

### Transgenic models

At least 10 genes have been identified as risk factors for PSP and 15 *MAPT* mutations are associated with the disease (Wen et al., 2021). Of these, the P301L mutation, first associated with frontotemporal dementia and parkinsonism linked to chromosome 17, has been most extensively utilized to generate transgenic mice. These animals develop a rapid and reliable tau pathology, which can be manipulated using different promoters and tau isoforms (Denk and Wade-Martins, 2007). However, the translational value of studies in these animals to PSP is hampered by several aspects. First, in the original description of tau pathology in patients bearing the P301L mutation, the pathology was described as comprising “Pick-like bodies”, which differ significantly from that described in PSP (Spillantini et al., 1998). Second, the P301L residue is located deep within the filament core of misfolded PSP tau (G272-N381) where it is unlikely a leucine residue can be accommodated, thus it is unlikely that P301L tau can replicate the filament structure associated with PSP (Shi et al., 2021).

Similarly, the A152T mutation in *MAPT* is a risk variant for many neurodegenerative proteinopathies and has been found in cases with symptoms consistent with PSP with pallido-nigro-luysial atrophy (Graff-Radford et al., 2013; Lee et al., 2013). A152T transgenic mice exhibit progressive aggregation of hyperphosphorylated tau in neurons of the hippocampus and cortex, synaptic loss, neurodegeneration and disrupted proteostasis with accompanying cognitive behavioral impairment (Sydow et al., 2016). Like their human counterpart, A152T mice also lack glial tau pathology (Kovacs et al., 2011), however they do exhibit a remarkable astrocytic and microglial neuroinflammatory involvement offering an important opportunity to study the relationship between tau accumulation and neuroinflammation.

Tau35 is a 35 kDa C-terminal cleavage product of tau that is highly aggregation prone and selectively found in the brains of patients with PSP, FTD and CBD (Wray et al., 2008; Lyu et al., 2021). Bondulich et al. (2016) developed a transgenic mouse that expresses human Tau35 under the control of the human tau promoter. These mice develop neuronal aggregates of hyperphosphorylated tau, predominantly in the hippocampus and cortex, impaired proteostasis and an age-related cognitive and locomotor phenotype, with no glial involvement. Notably, unlike many transgenic mice that rely upon overexpression of tau at levels not seen in human brain, Tau35 expression is equivalent to ~10% of mouse tau expression in these animals, offering a more physiological environment to study the effects of this tau fragment in disease pathogenesis.

Glial tau pathology, particularly the tufted astrocyte, is key characteristic feature of PSP. Higuchi et al. (2002) were among the first to attempt to recapitulate this pathology in transgenic mice by expressing tau in both neurons and glia using the Tα1 α-tubulin promoter. Encouragingly, although these animals express 3R tau, upon aging they developed Gallyas-positive oligodendroglial coiled bodies resembling those in PSP. However, the astrocytic pathology' was noted as being closer to that of CBD and there was surprising lack of neuronal pathology. Subsequently the same group generated a mouse that targeted astrocytes and expressed tau under the glial fibrillary acidic protein (GFAP) promoter (Forman et al., 2005). These animals developed a variety of astrocytic inclusions, including a subset, albeit in the minority, that bore a remarkable similarity to the tufted astrocytes that define PSP pathology.

Although mice have typically been the preferred species for the generation of tau transgenics, some tau transgenic rats do exist (Koson et al., 2008; Filipcik et al., 2010; Korhonen et al., 2009). Several lines have been generated that express a variety of truncated tau species in spontaneously hypertensive rats (SHR), these include the SHR72, SHR24 and SHR318 lines which all develop varying degrees of neurofibrillary tangle like pathology, neuronal loss and behavioral impairment (Koson et al., 2008; Filipcik et al., 2010; Zilka et al., 2006). However, the model with most relevance for PSP is the hTau-40/P301L rat, which develops mild deposition of hyperphosphorylated insoluble tau associated with dendritic abnormalities with no degeneration and minimal behavioral deficits, making them particularly appropriate for examining early “pretangle” pathology (Korhonen et al., 2009).

Although to date, there are no transgenic rodents that spontaneously develop the three key tau cytopathologies, behavioral phenotype or neurodegeneration seen in PSP, the mice generated to date do each offer an opportunity to study some critical aspect of the disease, be it related to specific genetic factors or an opportunity to selectively focus on neuronal, oligodendroglial or astrocytic pathology. Furthermore, transgenic mice have played an important role in PSP tau seeding studies, which we expand upon below. Importantly, most transgenic mouse lines are subject to caveats related to the spatial expression of tau being inextricably linked to the promoter used, as well as artifacts resulting from overexpression and genetic drift. However, the rapidly advancing technical acumen to develop ever more sophisticated models increases the potential for the future use of transgenic animals, as evidenced by the development of a mouse that expresses P301L tau labeled with green fluorescent protein in neurons, enabling the *in vivo* visualization of the propagation of tau pathology (Gibbons et al., 2017).

### Viral vector models

Adeno-associated virus (AAV)-based expression offers a sophisticated means to selectively express a protein in a given region of interest and even offers the opportunity to focus on a particular cell type using targeted promoters. This approach has been used extensively to study the role of tau in AD, and furthermore offers the potential to develop novel gene therapy-based treatments for tauopathies (Ittner et al., 2019). The vast majority of AAV-tau studies have focussed on the overexpression of P301L mutant tau, and for the same reasons noted above regarding P301L transgenic mice are thus of indirect value to the specific modeling of PSP. However, following reports identifying the A152T tau mutation as a risk factor for several tauopathies, including AD and PSP, Carlomangno *et al*. used somatic brain transgenesis to compare the effects of AAV-A152T and AAV-P301L tau in mouse brain (Carlomagno et al., 2019). While AAV-P301L resulted in development of insoluble hyperphosphorylated tau, in AAV-A152T mice hyperphosphorylated tau accumulated in an insoluble pool and was associated with distinct biochemical and neuroinflammatory effects coupled with astrocytosis and neuronal loss. Furthermore AAV-A152T mice developed a prominent locomotor behavioral phenotype, although this may be attributed to spinal cord pathology (Carlomagno et al., 2019).

We are aware of only a single study using AAV to specifically model PSP symptomatology. This recent work capitalized on a CRE-dependent AAV system to selectively express 1N4R human tau in the cholinergic neurons of the PPTg (King et al., 2021). Animals replicated several features of PSP including loss of ASR and a moderate locomotor deficit, along with reduction in both PPTg cholinergic neurons and nigral dopaminergic neurons accompanying the deposition of hyperphosphorylated tau. This model provides a platform to probe the role of PPTg cholinergic neurons in ASR and the development of early tau pathology, however, these animals do not encompass the glial contribution to PSP. Thus, long-term studies in these hTau rats are a critical next step to determine if a more aggressive PSP-like phenotype emerges over time.

### Inoculation studies in mice

The past decade has generated a wealth of evidence that neurodegenerative disease-associated proteins induce prion-like misfolding of endogenous healthy proteins (Ayers et al., 2020), with a key experimental paradigm being the inoculation of animal brains with human brain extracts bearing pathogenic seeds of the protein of interest (Robert et al., 2021). Importantly, the structure of misfolded tau has been shown to be distinct among the major tauopathies (Shi et al., 2021), yet to date only six inoculation studies in mice have used PSP tau, summarized in Table 1 (Narasimhan et al., 2020, 2017; Clavaguera et al., 2013; Xu et al., 2021; He et al., 2020; Ferrer et al., 2019).

**Table 1:** Summary of PSP human brain derived tau inoculation studies in mice.

**Reference**	**Donor demographics**		**Inoculum preparation**	**Mouse line (background, age at inoculation and N/timepoint)**	**Brain region(s) inoculated**	**Amount of inoculum per site**	**Endpoint (months post inoculation)**	**Astroglial pathology**	**Oligoden-droglial pathology**	**Neuronal pathology**	**Evidence of neurode-generation**	**Spread of pathology**
	**Brain region**	**Gender and age at death (years)**										
Clavaguera et al. (2013)	Putamen	M68, M72	PBS soluble and insoluble	ALZ17, 3 months, *N* = 5	Hippocampus and cerebral cortex	2.5 ul of 1:5 w/v PBS soluble and insoluble tau	6, 12 and 15	Present, region not described	Present, region not described	CA1 and CA2 hippocampal regions	None	Fimbria, optic tract, medial lemniscus, dorsal thalamus, cerebral peduncle, amygdala, internal capsule, entorhinal cortex, and fornix
				C57BL/6, 3 months, *N* = 6			6 and 15	None	Hippocampus			Optic tract, subiculum, and dorsal thalamus
Narasimhan et al. (2017)	Frontal Cortex, Thalamus and Lentiform	M74, F63, M65, M78, M71	1% Si	C57BL/6, 2-3 months, *N* = 2-4	Hippocampus and cerebral cortex,	0.7 ug tau/site; 2.5 ul	1, 3, 6 and 9	Hippocampus	Fimbria, corpus callosum	Hippocampus; dentate granule, hilar neurons, CA3, entorhinal cortex	None	Ventral hippocampus, entorhinal cortex, fimbria, dorsal hippocampus, olfactory bulb, corpus callosum, multiple cortical regions
					Thalamus	0.35 ug tau/site; 4 ul				Lateral posterior thalamic nucleus, dorsal raphe, superior colliculus, entorhinal cortex		Dorsal raphe, superior colliculus, entorhinal cortex, fimbria, hippocampus, visual cortex, anterior cingulate, prefrontal cortex, subiculum, mammillary area, midbrain nuclei, inferior colliculus, hypothalamus, other thalamic nuclei, prefrontal cortex
Ferrer et al. (2019)	Striatum	F72	0.1% Si	C57BL/6, 10–12 months, *N* = 3	Corpus callosum	1.2 ul/site	16–18	None	Corpus collosum	None	Slight myelin disruption and presence of small globules and balls in ipsilateral corpus callosum	Ipsilateral, middle, and contralateral corpus callosum
Narasimhan et al. (2020)	Frontal cortex	F63	1% Si	TauKDn^cre;fl/fl^, *N* = 4	Hippocampus and cerebral cortex	0.0371 ug/ul; 2.5 ul/site	3 and 6	Hippocampus	Fimbria, corpus callosum	Hippocampus	Not described	Contralateral fimbria, corpus callosum
				Tau^fl/fl^ *N* = 4								Contralateral fimbria, corpus callosum, hippocampus, multiple cortical regions
He et al. (2020)	Frontal cortex	M89, F63	1% Si	6hTau, 3-5 months, *N* = 3-6	Hippocampus and cerebral cortex	2.5 ul/site; 0.1-0.56ug/ul	1,3 and 6	Hippocampus	Fimbria	Hippocampus, multiple cortical regions	Not described	Entorhinal cortex, hippocampus, thalamus, hypothalamus, fimbria, corpus callosum, multiple cortical regions
Xu et al. (2021)	Mid Frontal Cortex	M72, F63, F72	1% Si	6hTau, 3–6 months, *N* = 4	Hippocampus	2 μg tau	3	Hippocampus	None	Hippocampus, multiple cortical regions	Not described	Entorhinal cortex, hypothalamus, thalamus, corpus callosum, multiple cortical regions

M, Male; F, Female; N, Number; Si, Sarkosyl insoluble.

**Figure 1:**
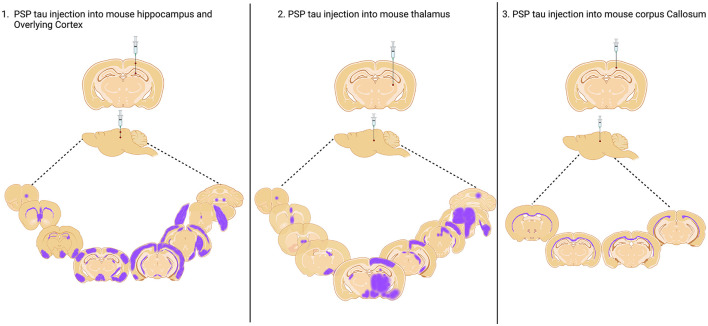
Injection sites of PSP Tau inoculation in nontransgenic mouse brain and aproximated reported spread to connected brain regions. Injection into the hippocampus and overlying cortex and thalamus as reported by Narasimhan et al. (2017), and into the Corpus Callosum as reported by Ferrer et al. (2019). Created with BioRender.com.

In a seminal study, Clavaguera and colleagues inoculated PBS soluble tau preparations from human PSP putamen into the hippocampus and overlying cortex of both nontransgenic and ALZ17 mice (Clavaguera et al., 2013). Over the following 6–15 months, all animals developed widespread silver positive tau aggregates in interconnected brain regions, including neurofibrillary tangles, oligodendroglial coiled bodies and astrocytic inclusions that closely resembled the tufted astrocytes characteristic of PSP. Following this, two further studies inoculated human PSP brain derived sarkosyl insoluble tau into wildtype mice. In the first study, they found neuronal inclusions as well as coiled bodies and astrocytic tau that propagated, by incorporation of endogenous mouse tau, via the neuronal connectome to regions distal to the hippocampal inoculation site (Narasimhan et al., 2017). Furthermore, the distribution and spread of tau pathology varied when two different inoculation sites (hippocampus and thalamus) were compared. The second study noted the presence of tau deposits predominantly in oligodendrocytes of the inoculated corpus callosum with little evidence of neuronal pathology (Ferrer et al., 2019). These contrasting studies highlight the likely importance of the site of inoculation on the cytopathologic deposition of tau and subsequent spread of pathology represented in Figure 1. In 2020, Narasimhan and colleagues inoculated mice lacking neuronal tau, finding that oligodendroglial tau pathology, but not astrocytic tau pathology was able to propagate in the absence of neuronal tau (Narasimhan et al., 2020). Finally, two recent inoculation studies have been performed in 6hTau mice, a pioneering transgenic model, which expresses an equal ratio of 3R and 4R human tau isoforms, akin to tau expression in the human brain (He et al., 2020). In these mice, hippocampal inoculation of sarkosyl insoluble human brain derived PSP tau generated all three PSP-related cytopathologies as early as 1 month post inoculation, with spread to the contralateral hemisphere at 6 months post inoculation (Xu et al., 2021; He et al., 2020). Importantly, the pathology induced is composed wholly of human tau isoforms offering exceptional translational value. Furthermore, the use of human derived seeds in mice expressing human tau offers the opportunity to more faithfully model pathogenic processing involved in the seeding of tau pathology in PSP.

While these excellent studies have provided important evidence confirming the ability of PSP-derived tau to induce the three cytopathologies that characterize PSP, as well as modeling the spread of pathology throughout interconnected regions of the brain, each of these studies were primarily focussed on other tauopathies, leading to gaps in experimental design with respect to the modeling of PSP which we elaborate on below.

### Studies in the non-human primate

Rhesus macaques (*Macaca mulatta*) are the phylogenetically closest animal to humans in which scientific research can be performed. As well as expressing 6 isoforms of tau, with the longest isoform sharing >98% sequence homology with the human counterpart, rhesus macaques have a human-like cerebral connectivity that offers an unparalleled opportunity to study cognitive and motor domains related to human disease. While this species is a gold standard for modeling Parkison's disease (Porras et al., 2012), only two studies have addressed the possibility to model PSP in them. In 2016, Uchihara et al. (2016) reported that although aged macaques develop Aβ-deposition reminiscent of Alzheimer's disease, intriguingly the distribution of tau was indicative of PSP pathology. Thus, in macaques aged >25 years, 4R tau-positive and Gallyas argyrophilic deposits mimicking PSP-like ultrastructure of filaments were found in oligodendroglia-like cells in the globus pallidus and white matter. Additionally, PSP-like tufted astrocytes were found in the temporal cortex and pre-tangle like structures were observed in the hippocampus. Furthermore, aged rhesus macaques developed increasing amounts of hyperphosphorylated insoluble 4R tau fragments with a molecular weight of 30–34kDa, similar to that which distinguishes PSP tau from other tauopathies (Taniguchi-Watanabe et al., 2016).

Recently, Darricau et al. (2023) performed the first PSP inoculation study infusing sarkosyl insoluble human PSP-tau in the supranigral region of two rhesus macaques. Over time both animals developed parkinsonism and dysexecutive syndromes. Furthermore, 18 months post inoculation, both animals developed glial and neuronal cytopathologies reminiscent of PSP, with evidence of nigral cell loss in a single animal. AT8 positive 4R-tau lesions were found in the supranigral area and ventral thalamus with spread to anteriorly connected regions including the caudate putamen, globus pallidus and thalamus. Morphological analysis revealed the presence of cytopathologic hallmarks of PSP, specifically, globose tangles, neurofibrillary tangles, oligodendroglial coiled bodies and tufted astrocytes. While astroglial lesions were found closest to injection site, consistent with a possible role of astroglia in sequestering tau (Narasimhan et al., 2020; Reid et al., 2020), the predominance of neuronal tau in interconnected brain regions was suggested to underscore transneuronal spread as the primary conduit for spreading tau pathology.

As we have previously discussed (Qamar and Visanji, 2023), this pioneering study suggests the rhesus macaque might be a key species to investigate the role of neuronal and glial cells in spreading pathological tau as well as the complex relationship between the different cytopathologies, neurodegeneration and clinical symptoms in PSP.

## Discussion: gaps in current animal models of PSP

A critical barrier in the study of PSP is that no existing animal model replicates the anatomical and cytopathologic hallmarks, as well as the spatiotemporal spread of pathology and progressive neurodegeneration that characterize the disease. Thus, although much has been learned from the wealth of research performed to date, here we will review where the present state-of -the-art falls short in recapitulating key features of PSP and suggest strategies where translational value might be improved in future endeavors.

### Tau isoform expression

An ideal PSP model would be generated in an animal that exhibits tau expression as closely to that in human brain as feasible. The human brain expresses 3R and 4R tau isoforms in approximately equal ratio (Goedert, 2003), although in PSP and CBD the 1:1 ratio is shifted in favor of 4R isoforms (Takanashi et al., 2002; Chambers et al., 1999). Interestingly, wildtype rodents only express 4R tau (Takuma et al., 2003). To combat this, more than 20 years ago Andorfer et al. (2003) generated mice expressing all 6 isoforms of human tau, but the 1:1 3R:4R ratio was not maintained. More recently, He et al. (2020), generated an innovative mouse model expressing all 6 human tau isoforms with a preserved 1:1 ratio of 3R:4R, offering an opportunity to model tauopathies in a mouse brain closely akin to the human counterpart.

Though studies in higher order species are only feasible in specialist laboratories and are subject to significant ethical implications, clearly the rhesus macaque offers many advantages in modeling human disease. With respect to modeling PSP pathology, a recent study has demonstrated the widespread presence of both 3R and 4R tau in macaque brain, including regions implicated in PSP (Gambardella et al., 2023).

### Spatiotemporal cytopathologic diversity

Inoculation studies have elegantly demonstrated that it is feasible to induce all three PSP relevant cytopathologies in the mouse brain (Narasimhan et al., 2020, 2017; Clavaguera et al., 2013; Xu et al., 2021; He et al., 2020; Ferrer et al., 2019). However, to date, none have modeled the reported regional diversity in tau cytopathology in PSP (Kovacs et al., 2020). Studies to date have inoculated the hippocampus, cortex, thalamus or corpus callosum and tau pathology has not been well described in the CPu, SN and GP (Table 1), the key subcortical brain regions associated with early PSP. Comparing PSP inoculation studies does provide helpful insight that the region inoculated appears to influence the predominant cytopathology seen (Table 1). Thus, animals inoculated in the corpus callosum exhibit oligodendroglial pathology (Ferrer et al., 2019), whereas neuronal pathology predominates in animals inoculated in the hippocampus (Xu et al., 2021; He et al., 2020). Furthermore, the site of inoculation likely influences the spread of tau pathology throughout the brain, and while several studies successfully observe spread (Table 1 and Figure 1), none describe this phenomenon among brain regions affected in PSP (Kovacs et al., 2020; Stamelou et al., 2021).

Modeling the tufted astrocyte, a key hallmark of PSP (Yoshida, 2014), also appears to pose a particular challenge, with the numbers seen reportedly scarce (Clavaguera et al., 2013; He et al., 2020). There is increasing appreciation of the importance of astrocytes in PSP (Kovacs, 2020). Some studies have suggested that astrocytes play a role in the sequestration of tau (Narasimhan et al., 2020). However, a recent study reported that, in addition to neurons, *MAPT* mRNA is also expressed in oligodendrocytes and astrocytes, and this expression is maintained in cells bearing aggregated tau in PSP (Forrest et al., 2023). Furthermore, in PSP inoculated macaques, Darricau et al. (2023) noted a locomotor phenotype, in the absence of significant striatal dopamine deficit, which instead was accompanied by strong astrocytic pathology in the peri-nigral region (Darricau et al., 2023). This points to the potential involvement of glial cells in proliferating tau aggregation and cellular dysfunction, rendering the presence of tufted astrocytes critical to capture underlying mechanisms of PSP.

### Behavioral phenotype and neurodegeneration

For optimal utility in testing of future therapies a model of PSP would ideally exhibit some degree of neurodegeneration, in pertinent regions of the brain, with an accompanying behavioral phenotype that captures the core clinical features of the disease. Modeling phenotypic variability in the clinical presentation of PSP poses a challenge, however, almost all cases initially present with motor features (Price and Clissold, 1989) and all subtypes eventually present as PSP-RS. It's therefore advisable that models focus on the key overlapping motor features of postural instability and akinesia as well as axial predominant parkinsonism.

To date, inoculation studies in mice have failed to report any neurodegeneration or behavioral impairments (Table 1). This might reflect in part the region inoculated, as well suggest that the degree of pathology induced, at least at the timepoints studied, is insufficient to induce neurodegeneration. Furthermore, the lack of overt cell loss might suggest that inoculation-induced pathology alone is itself not sufficient and that other pathogenic factors are required to induce neurodegeneration, or indeed that the limited lifespan of a mouse precludes the development of the severe neurodegenerative events associated with human PSP, which occurs over several years in later life (Painous et al., 2020).

PPTg lesion studies in rats have successfully selectively modeled ASR, a very specific phenotype (MacLaren et al., 2014b; Cyr et al., 2014). However, further studies found that, while spontaneous locomotion remained unaffected in PPTg lesioned animals, when challenged, a locomotor deficit attributed to nigral dopaminergic loss can be revealed, suggesting this model may offer additional opportunity for modeling PSP-related behaviors (MacLaren et al., 2018).

The non-human primate clearly offers enormous potential to model anthropomorphic locomotor and cognitive features inherent to PSP. However, it is noteworthy that the first PSP inoculation study performed in two Rhesus macaques found that only one animal had evidence of mild neurodegeneration (Darricau et al., 2023). Thus, future studies, employing a larger number of animals, will be needed to refine these challenging methodologies and generate the robust and replicable response to inoculation required to fully capitalize on the great promise of this species.

### PSP related seeding material for inoculation studies

Inoculation studies clearly hold great promise for modeling key cytopathologies in PSP, however there are many challenges in performing these experiments related to the preparation and use of the required inoculum. To date, the majority of these studies have relied on tau derived from PSP post-mortem brains. This poses several difficulties with respect to generating a robust, replicable and scalable model of PSP. First and foremost, only a limited number of groups have access to the well characterized human brain material required. Second, PSP is a rare disease and even large brain banks may only have a small number of PSP brains. Indeed, to date, only 10 different PSP cases have been used worldwide in inoculation studies (Table 1). Third, PSP is a remarkably heterogenous disease both clinically, pathologically and at a molecular level (Höglinger et al., 2017; Kovacs et al., 2020; Martinez-Valbuena et al., 2023), thus material needs to be comprehensively characterized by multidisciplinary teams comprised of movement disorder specialist neurologists, neuropathologists, and biochemists. Finally, the occurrence of concomitant pathologies in PSP brains is frequent (Rigby et al., 2015; Forrest and Kovacs, 2023) and, while some studies report the absence of concomitant pathologies in donor material (Xu et al., 2021; Ferrer et al., 2019), others do not address this (Narasimhan et al., 2020, 2017; Clavaguera et al., 2013; He et al., 2020).

A further challenge is posed by the yield of seeding competent tau from the human brain. Indeed, studies report using large masses of human brain, or even whole cortical regions to extract sufficient tau to inoculate only a modest number of animals (Table 1). Furthermore, Xu et al. (2021) report that the yield of insoluble tau from PSP brain is much lower than that of other tauopathies and can also vary up to 100-fold between PSP cases. We have recently found that the yield of tau from PSP post-mortem brain is heavily influenced by the extraction method employed, with tau yield decreasing as increasing concentrations of sarkosyl are used (Qamar et al., 2024). Studies have used a variety of methods to extract tau from human PSP brains, including PBS as well as varying percentages of sarkosyl (0.1–2%) (Clavaguera et al., 2013; Narasimhan et al., 2020, 2017; Xu et al., 2021; He et al., 2020; Ferrer et al., 2019). While sarkosyl is a useful reagent for isolating tau aggregates across a range of neurodegenerative diseases, it's established that sarkosyl isolation is not comprehensive for all tau isoforms present in the brain due to differences in the biochemical properties, phosphorylation and aggregation states of tau. Indeed, this has been clearly demonstrated both for PSP (Xiong et al., 2001) and AD brain extracts (Mukherjee et al., 2023). Given this, our observation, that altering the percentage of sarkosyl effects the efficiency of tau seeding *in vivo* (Qamar et al., 2024), raises the intriguing possibility that the use of different percentages of sarkosyl might alter the relative amounts of different isoforms of tau isolated, and that perhaps the extraction method can be optimized to capture the major tau species that are crucial for disease pathogenesis. Finally, the application of different sonication protocols can introduce further complexity to the assembly of tau species used for inoculation.

Evidently, the availability of a reliable, reproducible, and sustainable resource of PSP relevant seeding competent material would be of enormous benefit to the field. This critical need is being actively addressed in a variety of manners. Seeding-based amplification assays can be employed to amplify human tau strains *in vitro*, using recombinant tau as a substrate, with the resulting recombinant seeds having shared biophysical properties with the original human brain derived seed (Xu and Lee, 2023). Importantly, when the resulting amplified product was inoculated into 6hTau mice, animals developed both neurofibrillary tangles and tufted astrocytes, demonstrating that the recombinant seeds retained specific characteristics of the diseased starting material (Xu et al., 2021). The ability to generate infinite quantities of seeding competent material able to faithfully replicate disease specific features would revolutionize the future modeling of PSP. However, given the challenges reported regarding both the yield and purity of PSP tau influencing the amplification efficiency, it remains to be seen if this amplification methodology can be replicated in other laboratories. Furthermore, the authors note that the amplified tau could not perform similarly in a second cycle of *in vitro* amplification, hypothesizing that important cofactors might be diluted in successive rounds of *in vitro* amplification. A second approach that might be capitalized upon to address this need involves the cellular amplification PSP brain derived seeds. A recent study seeded Tau4RD^*^LM-YFP biosensor cells with PSP brain-derived tau seeds for 4 days to induce seeding of the endogenous tau (Zeng et al., 2023). Infected cells were then used to generate a monoclonal cell line that was shown to stably propagate misfolded tau. Furthermore, when cell lysates were used to seed recombinant tau *in vitro*, a population of the resulting fibrils were shown to have shared structural features with the original human brain derived seed. Although this study did not report on the amounts of misfolded tau generated by these monoclonal lines, this does provide exciting proof of principal that cultured cells can generate tau fibrils that faithfully replicate some structural features of the originating material. While very encouraging, it should be noted that Tau4RD^*^LM-YFP biosensor cells currently present some limitations when used to propagate PSP tau assemblies. First, as discussed above, the presence of the P301L mutation likely hinders the accurate modeling of PSP tau filament structures. Second, the V337M mutation, which is not typically found in PSP patients, could potentially impact the structure of the tau filaments. Third, the inclusion of a fluorescent tag in the biosensor system might interfere with the normal assembly and function of tau filaments. Finally, these cells express a tau construct containing only the 4-repeat domain (4R) from Q244 to N368 such that the biosensor does not fully encompass the complete core structure of PSP tau filaments which extend to residue N381.

These *in vitro* and cell-based amplification strategies are in their infancy, thus their true potential is unknown. While clearly able to amplify minuscule amounts of disease-associated tau seed, and early indications are that some degree of disease-related pathogenicity is preserved in the amplified material, it is important to note that the *in vitro* environment is likely lacking in critical cofactors present in brain (Xu et al., 2021). These might include those contributing to the post translational modifications (PTMs) of tau, that might be important to capture the full complexity of disease associated assemblies (Kyalu Ngoie Zola et al., 2023). That said, there are early indications that some cell-based approaches might conserve certain disease associated PTMs (Tarutani et al., 2023) making this an area of great promise.

## Conclusions and future directions

**Figure 2:**
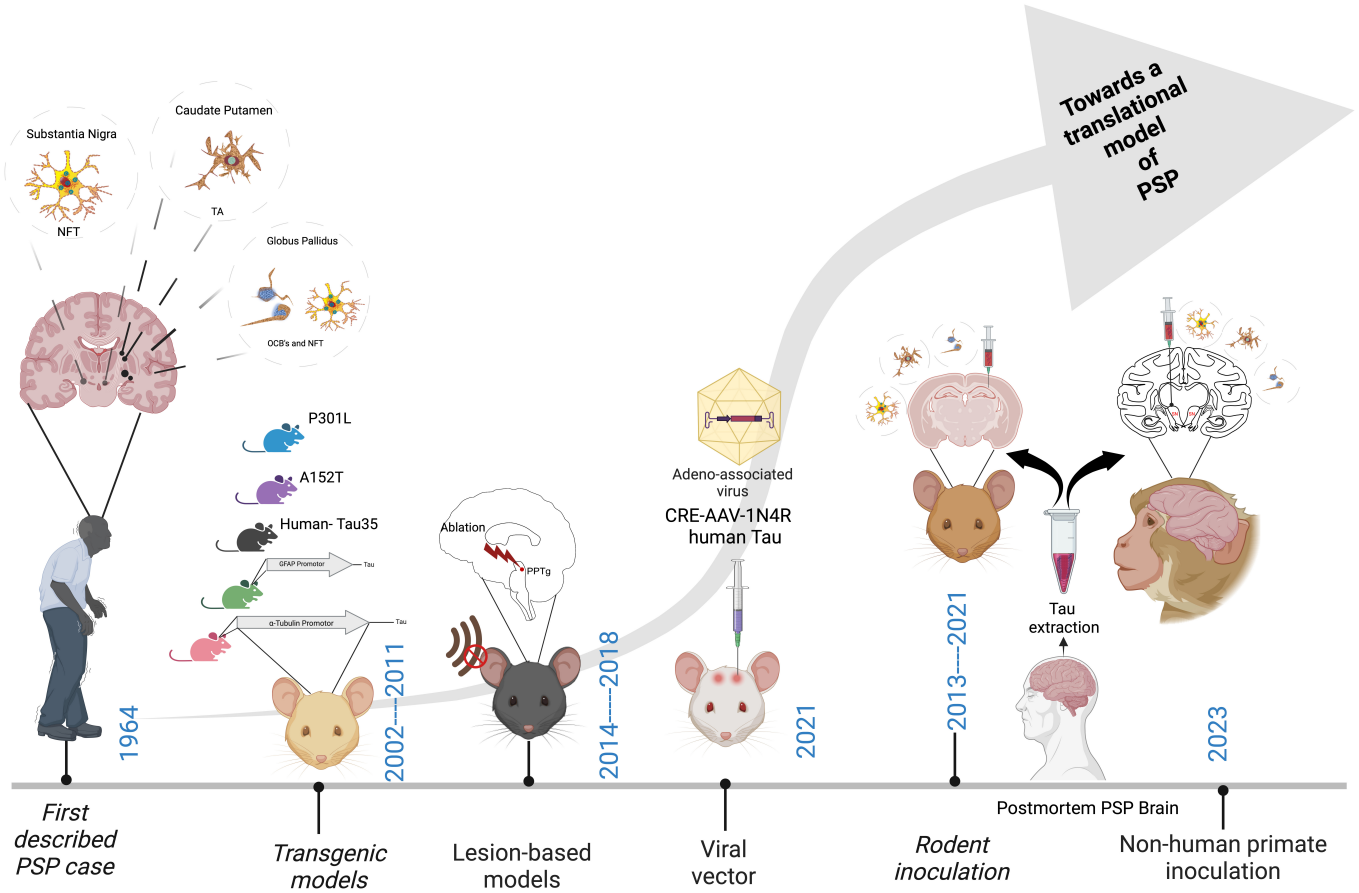
Major milestones in the development of an animal model of PSP. Neurofibrillary tangles (NFT), Tufted astrocytes (TA's) and Oligodendroglial coiled bodies (OCB's). Created with BioRender.com.

As Figure 2 depicts, since the first description of the disease in 1964 (Steele, 1964), much has been learned about PSP from the innovative studies performed in animals to date. These studies encompass a range of methodologies including transgenic rodents, lesion-based studies in rats, viral-vector mediated models as well as the more recent inoculation-based studies in both mice and non-human primates. However, an animal model that truly replicates the fundamental mechanisms of PSP pathogenesis, propagation and neurodegeneration remains a lacuna in the field. Such a model would provide a platform to significantly advance our knowledge of the fundamental biological mechanisms of PSP, as well as improving translational value for the preclinical testing of novel therapeutics.

Thus far, models of PSP have largely focussed on the contribution of tau pathology to the disease. However, as our understanding of the pathogenesis of PSP expands, so too do the desirable features of future animal models which should attempt to incorporate features such as neuroinflammation for example Langworth-Green et al. (2023) to optimize translational value. The use of an ever-expanding armory of state-of-the art tools will doubtless prove invaluable in this endeavor and facilitate judicious validation of models against the human disease. For example, recently, whole transcriptome spatial analysis identified a subset of cortical neurons vulnerable to Lewy pathology which were found to have a “conserved signature of molecular dysfunction” in both humans and a mouse model of Lewy body disease (Goralski et al., 2024). The use of sensitive tau seeding amplification assays (Saijo et al., 2019), as well as imaging in live animals (Wu et al., 2018; Ni, 2021) can be employed to enhance our capacity to study disease propagation throughout the brain *in vivo*.

Importantly, many of the concepts discussed in this review also have the potential for a broader impact in optimizing the way all tauopathies are modeled *in vivo* including, but not limited to, corticobasal degeneration, Pick's disease, argyrophilic grain disease and globular glial tauopathy. We remain optimistic that future endeavors, employing new technologies in refined animal models of PSP, will enrich our understanding of disease pathogenesis, allow the field to fully capitalize on the appealing translational value that PSP possesses among the tauopathies and enhance the development and testing of desperately needed disease modifying therapies.

## References

[B1] Andorfer C. Kress Y. Espinoza M. de Silva R. Tucker K. L. Barde Y-. A.. (2003). Hyperphosphorylation and aggregation of tau in mice expressing normal human tau isoforms. J. Neurochem. 86, 582–90. 10.1046/j.1471-4159.2003.01879.x 12859672

[B2] Arima K. (2006). Ultrastructural characteristics of tau filaments in tauopathies: Immuno-electron microscopic demonstration of tau filaments in tauopathies. Neuropathology 26, 475–483. 10.1111/j.1440-1789.2006.00669.x 17080728

[B3] Ayers J. I. Paras N. A. Prusiner S. B. (2020). Expanding spectrum of prion diseases. Emerg. Top Life Sci. 4, 155–167. 10.1042/ETLS20200037 32803268

[B4] Barer Y. Chodick G. Cohen R. Grabarnik-John M. Ye X. Zamudio J.. (2022). Epidemiology of progressive supranuclear palsy: real world data from the second largest health plan in Israel. Brain Sci. 12:1126. 10.3390/brainsci12091126 36138862 PMC9496895

[B5] Bondulich M. K. Guo T. Meehan C. Manion J. Rodriguez Martin T. Mitchell J. C.. (2016). Tauopathy induced by low level expression of a human brain-derived tau fragment in mice is rescued by phenylbutyrate. Brain 139, 2290–306. 10.1093/brain/aww137 27297240 PMC4958900

[B6] Carlomagno Y. Chung D-. E. C. Yue M. Kurti A. Avendano N. M. Castanedes-Casey M.. (2019). Enhanced phosphorylation of T153 in soluble tau is a defining biochemical feature of the A152T tau risk variant. Acta Neuropathol Commun 7:10. 10.1186/s40478-019-0661-2 30674342 PMC6345061

[B7] Chambers C. B. Lee J. M. Troncoso J. C. Reich S. Muma N. A. (1999). Overexpression of four-repeat tau mRNA isoforms in progressive supranuclear palsy but not in Alzheimer's disease. Ann. Neurol. 46, 325–332. 10482263 10.1002/1531-8249(199909)46:3<325::aid-ana8>3.0.co;2-v

[B8] Clavaguera F. Akatsu H. Fraser G. Crowther R. A. Frank S. Hench J.. (2013). Brain homogenates from human tauopathies induce tau inclusions in mouse brain. Proc. Natl. Acad. Sci. 110, 9535–9540. 10.1073/pnas.1301175110 23690619 PMC3677441

[B9] Cyr M. Parent M. J. Mechawar N. Rosa-Neto P. Soucy J-. P. Clark S. D.. (2014). Deficit in sustained attention following selective cholinergic lesion of the pedunculopontine tegmental nucleus in rat, as measured with both post-mortem immunocytochemistry and in vivo PET imaging with [18F]fluoroethoxybenzovesamicol. Behav. Brain Res. 278, 107–114. 10.1016/j.bbr.2014.09.021 25257103

[B10] Darricau M. Katsinelos T. Raschella F. Milekovic T. Crochemore L. Li Q.. (2023). Tau seeds from patients induce progressive supranuclear palsy pathology and symptoms in primates. Brain 146, 2524–2534. 10.1093/brain/awac428 36382344 PMC10232263

[B11] Denk F. Wade-Martins R. (2007). Knock-out and transgenic mouse models of tauopathies. Neurobiol. Aging 30, 1–13. 10.1016/j.neurobiolaging.2007.05.010 17590238 PMC2806682

[B12] Dujardin S. Commins C. Lathuiliere A. Beerepoot P. Fernandes A. R. Kamath T. V.. (2020). Tau molecular diversity contributes to clinical heterogeneity in Alzheimer's disease. Nat. Med. 26, 1256–1263. 10.1038/s41591-020-0938-9 32572268 PMC7603860

[B13] Ferrer I. García M. A. Carmona M. Andrés-Benito P. Torrejón-Escribano B. Garcia-Esparcia P.. (2019). Involvement of oligodendrocytes in tau seeding and spreading in tauopathies. Front. Aging Neurosci. 11:112. 10.3389/fnagi.2019.00112 31191295 PMC6546889

[B14] Filipcik P. Zilka N. Bugos O. Kucerak J. Koson P. Novak P.. (2010). First transgenic rat model developing progressive cortical neurofibrillary tangles. Neurobiol. Aging 33, 1448–1456. 10.1016/j.neurobiolaging.2010.10.015 21196063

[B15] Forman M. S. Lal D. Zhang B. Dabir D. V. Swanson E. Lee V. M-. Y.. (2005). Transgenic mouse model of tau pathology in astrocytes leading to nervous system degeneration. J. Neurosci. 25, 3539–50. 10.1523/JNEUROSCI.0081-05.2005 15814784 PMC6725385

[B16] Forrest S. L. Kovacs G. G. (2023). Current concepts of mixed pathologies in neurodegenerative diseases. Can. J. Neurol. Sci. 50, 329–345. 10.1017/cjn.2022.34 35356856

[B17] Forrest S. L. Lee S. Nassir N. Martinez-Valbuena I. Sackmann V. Li J.. (2023). Cell-specific MAPT gene expression is preserved in neuronal and glial tau cytopathologies in progressive supranuclear palsy. Acta Neuropathol. 146, 395–414. 10.1007/s00401-023-02604-x 37354322 PMC10412651

[B18] Gambardella J. C. Schoephoerster W. Bondarenko V. Yandell B. S. Emborg M. E. (2023). Expression of tau and phosphorylated tau in the brain of normal and hemiparkinsonian rhesus macaques. J. Comp. Neurol. 531, 1198–1216. 10.1002/cne.25490 37098996 PMC10247506

[B19] Gibbons G. S. Banks R. A. Kim B. Xu H. Changolkar L. Leight S. N.. (2017). GFP-mutant human tau transgenic mice develop tauopathy following CNS injections of Alzheimer's brain-derived pathological tau or synthetic mutant human tau fibrils. J. Neurosci. 37, 11485–11494. 10.1523/JNEUROSCI.2393-17.2017 28986461 PMC5700428

[B20] Goedert M. (2003). Tau protein and neurodegeneration. Semin Cell Dev. Biol. 15, 45–9. 10.1016/j.semcdb.2003.12.015 15036206

[B21] Goedert M. Crowther R. A. Scheres S. H. W. Spillantini M. G. (2024). Tau and neurodegeneration. Cytoskeleton 81:21812. 10.1002/cm.21812 38073060 PMC7615684

[B22] Goedert M. Eisenberg D. S. Crowther R. A. (2017). Propagation of tau aggregates and neurodegeneration. Annu. Rev. Neurosci. 40, 189–210. 10.1146/annurev-neuro-072116-031153 28772101

[B23] Goralski T. M. Meyerdirk L. Breton L. Brasseur L. Kurgat K. DeWeerd D.. (2024). Spatial transcriptomics reveals molecular dysfunction associated with cortical Lewy pathology. Nat. Commun. 15:2642. 10.1038/s41467-024-47027-8 38531900 PMC10966039

[B24] Graff-Radford J. Whitwell J. L. Dickson D. W. Josephs K. A. (2013). Pallidonigroluysian atrophy associated with p.A152T variant in MAPT. Parkinsonism Relat. Disord. 19, 838–41. 10.1016/j.parkreldis.2013.04.023 23692670

[B25] He Z. McBride J. D. Xu H. Changolkar L. Kim S. Zhang B.. (2020). Transmission of tauopathy strains is independent of their isoform composition. Nat. Commun. 11:7. 10.1038/s41467-019-13787-x 31911587 PMC6946697

[B26] Higuchi M. Ishihara T. Zhang B. Hong M. Andreadis A. Trojanowski J.. (2002). Transgenic mouse model of tauopathies with glial pathology and nervous system degeneration. Neuron 35, 433–46. 10.1016/S0896-6273(02)00789-4 12165467

[B27] Hirsch E. C. Graybiel A. M. Duyckaerts C. Javoy-Agid F. (1987). Neuronal loss in the pedunculopontine tegmental nucleus in Parkinson disease and in progressive supranuclear palsy. Proc. Natl. Acad. Sci. 84, 5976–80. 10.1073/pnas.84.16.5976 3475716 PMC298986

[B28] Höglinger G. Respondek G. Stamelou M. Kurz C. Josephs K. Lang A.. (2017). Movement disorder society - clinical diagnostic criteria for progressive supranuclear palsy. Mov. Disord. 32, 853–864. 10.1002/mds.26987 28467028 PMC5516529

[B29] Hyman B. (2023). All the tau we cannot see. Annu. Rev. Med. 74, 503–514. 10.1146/annurev-med-042921-023749 36378913 PMC11783578

[B30] Ittner L. M. Klugmann M. Ke Y. D. (2019). Adeno-associated virus-based Alzheimer's disease mouse models and potential new therapeutic avenues. Br. J. Pharmacol. 176, 3649–3665. 10.1111/bph.14637 30817847 PMC6715621

[B31] Jellinger K. (1988). The pedunculopontine nucleus in Parkinson's disease, progressive supranuclear palsy and Alzheimer's disease. J. Neurol. Neurosurg. Psychiatry 51, 540–3. 10.1136/jnnp.51.4.540 3379428 PMC1032970

[B32] King G. Veros K. M. MacLaren D. A. A. Leigh M. P. K. Spernyak J. A. Clark S. D.. (2021). Human wildtype tau expression in cholinergic pedunculopontine tegmental neurons is sufficient to produce PSP-like behavioural deficits and neuropathology. Eur. J. Neurosci. 54, 7688–7709. 10.1111/ejn.15496 34668254 PMC9355171

[B33] Korhonen P. van Groen T. Thornell A. Kyrylenko S. Soininen M-. L. Ojala J.. (2009). Characterization of a novel transgenic rat carrying human tau with mutation P301L. Neurobiol. Aging 32, 2314–2315. 10.1016/j.neurobiolaging.2009.12.022 20097445

[B34] Koson P. Zilka N. Kovac A. Kovacech B. Korenova M. Filipcik P.. (2008). Truncated tau expression levels determine life span of a rat model of tauopathy without causing neuronal loss or correlating with terminal neurofibrillary tangle load. Eur. J. Neurosci. 28, 239–46. 10.1111/j.1460-9568.2008.06329.x 18702695

[B35] Kovacs G. G. (2020). Astroglia and tau: new perspectives. Front. Aging Neurosci. 12:96. 10.3389/fnagi.2020.00096 32327993 PMC7160822

[B36] Kovacs G. G. Lukic M. J. Irwin D. J. Arzberger T. Respondek G. Lee E. B.. (2020). Distribution patterns of tau pathology in progressive supranuclear palsy. Acta Neuropathol. 140, 99–119. 10.1007/s00401-020-02158-2 32383020 PMC7360645

[B37] Kovacs G. G. Wöhrer A. Ströbel T. Botond G. Attems J. Budka H.. (2011). Unclassifiable tauopathy associated with an A152T variation in MAPT exon 7. Clin. Neuropathol. 30, 3–10. 10.5414/NPP30003 21176711

[B38] Kyalu Ngoie Zola N. Balty C. Pyr Dit Ruys S. Vanparys A. A. T. Huyghe N. D. G. Herinckx G.. (2023). Specific post-translational modifications of soluble tau protein distinguishes Alzheimer's disease and primary tauopathies. Nat. Commun. 14:3706. 10.1038/s41467-023-39328-1 37349319 PMC10287718

[B39] Langworth-Green C. Patel S. Jaunmuktane Z. Jabbari E. Morris H. Thom M.. (2023). Chronic effects of inflammation on tauopathies. Lancet Neurol. 22, 430–442. 10.1016/S1474-4422(23)00038-8 37059510

[B40] Lee S. E. Tartaglia M. C. Yener G. Genç S. Seeley W. W. Sanchez-Juan P.. (2013). Neurodegenerative disease phenotypes in carriers of MAPT p.A152T, a risk factor for frontotemporal dementia spectrum disorders and Alzheimer disease. Alzheimer Dis. Assoc. Disord. 27, 302–9. 10.1097/WAD.0b013e31828cc357 23518664 PMC3796183

[B41] Lyu C. Da Vela S. Al-Hilaly Y. Marshall K. E. Thorogate R. Svergun D.. (2021). The Disease associated tau35 fragment has an increased propensity to aggregate compared to full-length tau. Front. Mol. Biosci. 8:779240. 10.3389/fmolb.2021.779240 34778381 PMC8581542

[B42] MacLaren D. A. A. Ljungberg T. L. Griffin M. E. Clark S. D. (2018). Pedunculopontine tegmentum cholinergic loss leads to a progressive decline in motor abilities and neuropathological changes resembling progressive supranuclear palsy. Eur. J. Neurosci. 48, 3477–3497. 10.1111/ejn.14212 30339310

[B43] MacLaren D. A. A. Markovic T. Clark S. D. (2014a). Assessment of sensorimotor gating following selective lesions of cholinergic pedunculopontine neurons. Eur. J. Neurosci. 40, 3526–37. 10.1111/ejn.12716 25208852

[B44] MacLaren D. A. A. Santini J. A. Russell A. L. Markovic T. Clark S. D. (2014b). Deficits in motor performance after pedunculopontine lesions in rats–impairment depends on demands of task. Eur. J. Neurosci. 40, 3224–3236. 10.1111/ejn.12666 24995993

[B45] Majumder M. Dutta D. (2024). Oligodendrocyte dysfunction in tauopathy: a less explored area in tau-mediated neurodegeneration. Cells 13:1112. 10.3390/cells13131112 38994964 PMC11240328

[B46] Martinez-Valbuena I. Lee S. Santamaria E. Fernandez Irigoyen J. Li J. Tanaka H.. (2023). 4R-Tau seeding activity unravels molecular subtypes in patients with Progressive Supranuclear Palsy 2 3 4. bioRxiv [preprint]. 10.1101/2023.09.28.559953 37808843 PMC10557711

[B47] Mate de Gerando A. Quittot N. Frosch M. P Hyman B. T. (2023). Reply: soluble oligomers or insoluble fibrils? Acta Neuropathol. 146, 863–866. 10.1007/s00401-023-02634-5 37733036 PMC10628010

[B48] Mate De Gerando A. Welikovitch L. A. Khasnavis A. Commins C. Glynn C. Chun J. E.. (2023). Tau seeding and spreading in vivo is supported by both AD-derived fibrillar and oligomeric tau. Acta Neuropathol. 146, 191–210. 10.1007/s00401-023-02600-1 37341831 PMC10329061

[B49] Mukherjee S. Dubois C. Perez K. Varghese S. Birchall I. E. Leckey M.. (2023). Quantitative proteomics of tau and Aβ in detergent fractions from Alzheimer's disease brains. J. Neurochem. 164, 529–552. 10.1111/jnc.15713 36271678

[B50] Narasimhan S. Changolkar L. Riddle D. M. Kats A. Stieber A. Weitzman S. A.. (2020). Human tau pathology transmits glial tau aggregates in the absence of neuronal tau. J. Exp. Med. 217:e20190783. 10.1084/jem.20190783 31826239 PMC7041709

[B51] Narasimhan S. Guo J. L. Changolkar L. Stieber A. McBride J. D. Silva L. V.. (2017). Pathological tau strains from human brains recapitulate the diversity of tauopathies in nontransgenic mouse brain. J. Neurosci. 37, 11406–11423. 10.1523/JNEUROSCI.1230-17.2017 29054878 PMC5700423

[B52] Nath U. Ben-Shlomo Y. Thomson R. G. Morris H. R. Wood N. W. Lees A. J.. (2001). The prevalence of progressive supranuclear palsy (Steele-Richardson-Olszewski syndrome) in the UK. Brain 124, 1438–49. 10.1093/brain/124.7.1438 11408338

[B53] Ni R. (2021). Magnetic resonance imaging in tauopathy animal models. Front. Aging Neurosci. 13:791679. 10.3389/fnagi.2021.791679 35145392 PMC8821905

[B54] Painous C. Martí M. J. Simonet C. Garrido A. Valldeoriola F. Muñoz E.. (2020). Prediagnostic motor and non-motor symptoms in progressive supranuclear palsy: The step-back PSP study. Parkinsonism Relat. Disord. 74, 67–73. 10.1016/j.parkreldis.2020.03.003 32536421

[B55] Porras G. Li Q. Bezard E. (2012). Modeling Parkinson's disease in primates: the MPTP model. Cold Spring Harb. Perspect. Med. 2:a009308. 10.1101/cshperspect.a009308 22393538 PMC3282499

[B56] Price A. H. Clissold S. P. (1989). Salbutamol in the 1980s. A reappraisal of its clinical efficacy. Drugs 38, 77–122. 10.2165/00003495-198938010-00004 2670512

[B57] Qamar S. H. Ferry R. Mao A. Martinez-Valbuena I. Visanji N. (2024). “Optimization of tau extraction from human post-mortem progressive supranuclear palsy brain for modelling tau pathology *in vivo*,” in Proceedings of the 18th International Conference on Alzheimer's and Parkinson's Diseases (Lisbon: ADPD).

[B58] Qamar S. H. Visanji N. P. (2023). The MonKEY to unlocking the pathogenesis of progressive supranuclear palsy. Mov. Disord. 38, 953–954. 10.1002/mds.29404 37050873

[B59] Reid M. J. Beltran-Lobo P. Johnson L. Perez-Nievas B. G. Noble W. (2020). Astrocytes in Tauopathies. Front. Neurol. 11:572850. 10.3389/fneur.2020.572850 33071951 PMC7542303

[B60] Rigby H. B. Dugger B. N. Hentz J. G. Adler C. H. Beach T. G. Shill H. A.. (2015). Clinical features of patients with concomitant parkinson's disease and progressive supranuclear palsy pathology. Mov. Disord. Clin. Pract. 2, 33–38. 10.1002/mdc3.12104 30363831 PMC6183005

[B61] Robert A. Schöll M. Vogels T. (2021). Tau seeding mouse models with patient brain-derived aggregates. Int. J. Mol. Sci. 22:6132. 10.3390/ijms22116132 34200180 PMC8201271

[B62] Saijo E. Groveman B. R. Kraus A. Metrick M. Orrù C. D. Hughson A. G.. (2019). Ultrasensitive RT-QuIC seed amplification assays for disease-associated tau, α-synuclein, and prion aggregates. Methods Mol. Biol. 1873, 19–37. 10.1007/978-1-4939-8820-4_2 30341601

[B63] Shi Y. Zhang W. Yang Y. Murzin A. G. Falcon B. Kotecha A.. (2021). Structure-based classification of tauopathies. Nature 598, 359–363. 10.1038/s41586-021-03911-7 34588692 PMC7611841

[B64] Shoeibi A. Olfati N. Litvan I. (2019). Frontrunner in translation: progressive supranuclear palsy. Front. Neurol. 10:1125. 10.3389/fneur.2019.01125 31695675 PMC6817677

[B65] Spillantini M. G. Crowther R. A. Kamphorst W. Heutink P. Van Swieten J. C. (1998). Tau pathology in two Dutch families with mutations in the microtubule- binding region of tau. Am. J. Pathol. 153:1359–1363. 10.1016/S0002-9440(10)65721-5 9811325 PMC1853390

[B66] Stamelou M. Respondek G. Giagkou N. Whitwell J. L. Kovacs G. G. Höglinger G. U.. (2021). Evolving concepts in progressive supranuclear palsy and other 4-repeat tauopathies. Nat. Rev. Neurol. 17:5. 10.1038/s41582-021-00541-5 34426686

[B67] Steele J. C. (1964). Progressive supranuclear palsy. Arch. Neurol. 10:333. 10.1001/archneur.1964.00460160003001 14107684

[B68] Stern A. M. Selkoe D. J. (2023). Soluble oligomers or insoluble fibrils? Scientific commentary on “Tau seeding and spreading *in vivo* is supported by both AD-derived fibrillar and oligomeric tau”. Acta Neuropathol 146, 861–862. 10.1007/s00401-023-02633-6 37733037

[B69] Sydow A. Hochgräfe K. Könen S. Cadinu D. Matenia D. Petrova O.. (2016). Age-dependent neuroinflammation and cognitive decline in a novel Ala152Thr-Tau transgenic mouse model of PSP and AD. Acta Neuropathol. Commun. 4:17. 10.1186/s40478-016-0281-z 26916334 PMC4766625

[B70] Takanashi M. Mori H. Arima K. Mizuno Y. Hattori N. (2002). Expression patterns of tau mRNA isoforms correlate with susceptible lesions in progressive supranuclear palsy and corticobasal degeneration. Brain Res. Mol. Brain Res. 104, 210–219. 10.1016/S0169-328X(02)00382-0 12225876

[B71] Takigawa H. Kitayama M. Wada-Isoe K. Kowa H. Nakashima K. (2016). Prevalence of progressive supranuclear palsy in Yonago: change throughout a decade. Brain Behav. 6:e00557. 10.1002/brb3.557 28031995 PMC5166993

[B72] Takuma H. Arawaka S. Mori H. (2003). Isoforms changes of tau protein during development in various species. Brain Res. Dev. Brain Res. 142, 121–127. 10.1016/S0165-3806(03)00056-7 12711363

[B73] Taniguchi-Watanabe S. Arai T. Kametani F. Nonaka T. Masuda-Suzukake M. Tarutani A.. (2016). Biochemical classification of tauopathies by immunoblot, protein sequence and mass spectrometric analyses of sarkosyl-insoluble and trypsin-resistant tau. Acta Neuropathol. 131, 267–280. 10.1007/s00401-015-1503-3 26538150 PMC4713716

[B74] Tarutani A. Adachi T. Akatsu H. Hashizume Y. Hasegawa K. Saito Y.. (2022). Ultrastructural and biochemical classification of pathogenic tau, α-synuclein and TDP-43. Acta Neuropathol. 143, 613–640. 10.1007/s00401-022-02426-3 35513543 PMC9107452

[B75] Tarutani A. Hasegawa M. (2022). Role of structural polymorphisms of tau filaments in the pathological diversity of tauopathies. Brain Nerve 74, 919–925. 10.11477/mf.1416202150 35860941

[B76] Tarutani A. Kametani F. Tahira M. Saito Y. Yoshida M. Robinson A. C.. (2023). Distinct tau folds initiate templated seeding and alter the post-translational modification profile. Brain 146, 4988–4999. 10.1093/brain/awad272 37904205 PMC10690015

[B77] Uchihara T. Endo K. Kondo H. Okabayashi S. Shimozawa N. Yasutomi Y.. (2016). Tau pathology in aged cynomolgus monkeys is progressive supranuclear palsy/corticobasal degeneration- but not Alzheimer disease-like -Ultrastructural mapping of tau by EDX. Acta Neuropathol. Commun. 4:118. 10.1186/s40478-016-0385-5 27842611 PMC5109723

[B78] Wen Y. Zhou Y. Jiao B. Shen L. (2021). Genetics of progressive supranuclear palsy: a review. J. Parkinsons. Dis. 11, 93–105. 10.3233/JPD-202302 33104043 PMC7990399

[B79] Wray S. Saxton M. Anderton B. H. Hanger D. P. (2008). Direct analysis of tau from PSP brain identifies new phosphorylation sites and a major fragment of N-terminally cleaved tau containing four microtubule-binding repeats. J. Neurochem. 105, 2343–52. 10.1111/j.1471-4159.2008.05321.x 18315566

[B80] Wu Q. Lin Y. Gu J. Sigurdsson E. M. (2018). Dynamic assessment of tau immunotherapies in the brains of live animals by two-photon imaging. EBioMed. 35, 270–278. 10.1016/j.ebiom.2018.08.041 30146345 PMC6158769

[B81] Xiong L. W. Raymond L. D. Hayes S. F. Raymond G. J. Caughey B. (2001). Conformational change, aggregation and fibril formation induced by detergent treatments of cellular prion protein. J. Neurochem. 79, 669–678. 10.1046/j.1471-4159.2001.00606.x 11701770

[B82] Xu H. Lee V. M-. Y. (2023). *In vitro* amplification of pathogenic tau seeds from neurodegenerative disease patient brains. Methods Mol. Biol. 2561, 279–292. 10.1007/978-1-0716-2655-9_15 36399276

[B83] Xu H. O'Reilly M. Gibbons G. S. Changolkar L. McBride J. D. Riddle D. M.. (2021). In vitro amplification of pathogenic tau conserves disease-specific bioactive characteristicas. Acta Neuropathol. 141, 193–215. 10.1007/s00401-020-02253-4 33385254 PMC7847465

[B84] Yoshida M. (2014). Astrocytic inclusions in progressive supranuclear palsy and corticobasal degeneration. Neuropathology 34, 555–70. 10.1111/neup.12143 25124031

[B85] Zeng Z. Vijayan V. Tsay K. Frost M. P. Quddus A. Albert A.. (2023). PSP cell-passaged tau seeds generate heterogeneous fibrils with a sub-population adopting disease folds. bioRxiv [preprint]. 10.1101/2023.07.19.549721 37502998 PMC10370138

[B86] Zilka N. Filipcik P. Koson P. Fialova L. Skrabana R. Zilkova M.. (2006). Truncated tau from sporadic Alzheimer's disease suffices to drive neurofibrillary degeneration *in vivo*. FEBS Lett. 580, 3582–3588. 10.1016/j.febslet.2006.05.029 16753151

